# IgG-Dependent Dismutation of Superoxide in Patients with Different Types of Multiple Sclerosis and Healthy Subjects

**DOI:** 10.1155/2020/8171020

**Published:** 2020-02-05

**Authors:** Liudmila P. Smirnova, Irina A. Mednova, Nina M. Krotenko, Valentina M. Alifirova, Svetlana A. Ivanova

**Affiliations:** ^1^Laboratory of Molecular Genetics and Biochemistry, Mental Health Research Institute, Tomsk National Research Medical Center of the Russian Academy of Sciences, Tomsk 634014, Russia; ^2^Department of Neurology and Neurosurgery, Siberian State Medical University, Tomsk 644050, Russia

## Abstract

This work is the first to demonstrate that class G immunoglobulins (IgGs) in patients with multiple sclerosis and healthy individuals have the ability to catalyze the dismutation reaction of the superoxide anion radical. Thus, superoxide dismutase (SOD) activity is an intrinsic property of antibodies, which is confirmed by a number of stringent criteria. SOD activity of IgGs in patients with multiple sclerosis statistically significantly exceeds such activity in healthy individuals by 2-4 times. Moreover, the maximum activity has been registered in patients with relapsing remitting multiple sclerosis. The kinetic characteristics of the SOD reaction of IgGs are several orders of magnitude lower than those for the SOD enzyme but do not differ between patients with multiple sclerosis and healthy individuals. Consequently, abzymes with SOD activity have a lower catalysis rate than that of the enzymes and form a stronger complex with the substrates. Inhibitory analysis showed that this activity is inhibited by classical metal-dependent SOD inhibitors. The activity of IgGs was inhibited by classical metal-dependent inhibitors EDTA and TETA (triethylenetetramine). Also, high catalase activity of IgGs was detected in these patients. We suggest that these abzymes help protect the body from oxidative stress.

## 1. Introduction

Oxidative stress (OS) is one of the leading pathophysiological factors in the development of many central nervous system diseases including diseases as serious as multiple sclerosis (MS). Processes of inflammation and OS feed each other, and both play a significant role in the pathogenesis of MS.

The brain is susceptible to OS not only due to high oxygen saturation or increased content of easily oxidizable polyunsaturated fatty acids in myelin shells but also due to the low amount and activity of antioxidants present in the brain than in other tissues [1]. As a result, free radicals form in large quantities and react with many biological molecules, causing damage to various membranes, transcription factors, proteins, and DNA in oligodendrocytes and neurons [2–4]. Generalized OS occurring in MS is accompanied by an imbalance in the enzymatic and nonenzymatic components of the antioxidant defense system (AODS) [5–11]. Recent investigations have revealed reduced activity of antioxidant enzymes (AE) (superoxide dismutase, glutathione reductase), as well as decreased levels of glutathione, tocopherol, ubiquinone, transferrin, ascorbic acid, retinol, and thiols in the cerebrospinal fluid, plasma, and blood cells of patients with MS [8, 12–14].

Most researchers adhere to the concept of a two-phase model of MS [15–18]. The first phase is characterized by an inflammatory process with frequent exacerbations and remissions, which are accompanied by demyelination and the appearance of lesions on magnetic resonance imaging (MRI). The second phase is related to neurodegeneration. The specific antibodies against various components of myelin, lipid molecules, DNA, and other tissues can be detected in patients with MS [19]. The pathogenetic and clinical relevance of these antibodies has not been sufficiently studied. At an early stage of MS, macrophages strip myelin from axons and phagocytose myelin fragments, thereby blocking the conduction of nerve impulses. A reduced antioxidant reserve and generalized OS can possibly be the underlying causes of the second phase of the disease.

In MS, the remyelination process occurs in parallel with demyelination and includes regeneration of myelin by oligodendrocytes and axon branching with the formation of new synapses that replace the dead ones [20]. Under certain conditions, remyelination can be stimulated by antibodies (Abs) produced by B cells. One of the latest advancements in MS treatment is the use of remyelination-promoting Abs including synthetic ones [21]. In this respect, an important role is given to Abs possessing protease activity and capable of reconstructing damaged myelin in different areas of the nervous system [22, 23]. Thus, both T cells and B cells can play a dual role in the development of MS [24].

In this regard, of particular interest are the works on Abs with natural catalytic activity. In 1989, a group of researchers led by S. Paul first discovered IgGs with proteolytic activity in the blood serum of patients with bronchial asthma [25]. Abs possessing catalytic activity were called abzymes. Recently, a link between the abzymatic activity of autoAbs and neurodegenerative processes has been demonstrated [26, 27]. The phenomenon of immunoglobulins having catalytic properties in MS has been actively studied in recent decades. Catalytic Abs or abzymes with DNase, RNase, proteolytic, and amylolytic activities were found in the blood of patients with MS [28–31].

Such a variety of enzymatic activities of Abs suggests that natural Abs, while interacting with a large number of substrates, may allow the body to maintain a normal level of homeostasis. It is still unclear whether catalytic Abs are real effectors of the pathogenic immune response, or whether their formation is secondary to a disturbance in immune network homeostasis.

Catalytic antibodies with oxidoreductase activities have not been completely studied. There is insufficient information about the presence of catalytically active Abs possessing oxidoreductase activity in the blood serum of animals. In particular, monoclonal and polyclonal Abs of humans and other mammals were studied for a long time by Lerner's group, and it was proved that these Abs are able to interact with singlet oxygen and effectively recover it [32]. Kulberg et al. showed for the first time that IgGs obtained from rabbit blood catalyze superoxide-dependent processes [33]. The presence of catalytically active Abs with oxidoreductase activity in the serum of animals and healthy people was already discovered in the 21st century [34–36]. Ikhmyangan et al. showed that polyclonal Abs from the sera of Wistar rats possess oxidoreductase activity in the presence and absence of hydrogen hydroperoxide [37–39]. In addition, our recent studies conducted together with the Institute of Chemical Biology and Fundamental Medicine (Novosibirsk, Russia) have reported catalase (CAT) activity in IgGs of patients with schizophrenia [40]. The study of the oxidoreductase activity of IgGs has not previously been conducted in patients with MS. In this work, the catalytic properties of IgGs with superoxide dismutase activity and catalase activity were studied for the first time in patients with relapsing remitting MS (RRMS) and secondary progressive MS (SPMS).

## 2. Materials and Methods

### 2.1. Characteristics of the Studied Subjects

The experimental part of the work was performed in the Laboratory of Molecular Genetics and Biochemistry of the Research Institute of Mental Health at the Tomsk National Research Medical Center (TNRMC). The studied subjects, matched for age and sex, underwent a comprehensive clinical and biological examination and were under our supervision. The diagnosis of MS was confirmed by MRI and established by the doctors of the Department of Neurology and Neurosurgery of Siberian State Medical University using the McDonald diagnostic criteria for MS (2001, 2005). Patients with MS were divided into two groups according to disease course. The first group included 25 patients with RRMS. The second group comprised 20 patients with SPMS. The control group included 20 healthy subjects. The study was carried out according to the protocol approved by the Biomedicine Ethic Committee of Siberian State Medical University (Tomsk, Russia) and in accordance with Helsinki Declaration for human experimentation. Informed consent for participation in clinical research was obtained from all examined individuals.

The neurological status of patients was assessed by the Expanded Disability Status Scale (EDSS) [41]. Patients were in the acute stage of the disease and did not receive immunosuppressive therapy for at least 6 months before beginning the study.

The median age of patients with RRMS was 31 (21; 37) years, average age of onset of the disease was 26 ± 6 years, average disease duration was 6 ± 2 years, and average disability degree using the EDSS scale was 2.5 ± 1.5 points.

The median age of patients with SPMS was 47 (38; 65) years, average age of onset of the disease was 27 ± 7 years, average disease duration was 16 ± 5 years, and average disability degree using the EDSS scale was 4 ± 1.5 points.

The control group consisted of individuals without a history of somatic or psychiatric disorders. The selection of healthy individuals was carried out using in-depth interviews with specially designed “survey of healthy individuals.” The median age of control group was 37 (33; 50) years.

### 2.2. Object of Study

Peripheral blood serum from patients with MS and healthy individuals was used as the study material. Blood from the ulnar vein was taken from the examined subjects on an empty stomach in the morning. Vacutainer tubes with an activator of blood coagulation (inert silicon particles of SiO_2_ deposited on the inner walls of the tube) were used for blood collection. The test tube with blood was centrifuged at 2000g for 20 minutes at a temperature of +4°C in a refrigerated centrifuge Orto Alresa Digicen 21R (Spain) to separate blood serum from the form elements.

### 2.3. Purification of IgGs

The serum of patients and healthy individuals was diluted three times with buffer A (0.05 M Tris-HCl (pH 7.5) and 0.15 M NaCl). Then, the sample was applied to a Protein G Sepharose column (HiTrap™ Protein G HP, 1 ml) (GE Healthcare Bio-Sciences, Sweden) using ÄKTA pure chromatography system (GE Healthcare Bio-Sciences, Sweden). The column was washed with buffer A to zero optical density (A280). Nonspecific adsorbed proteins and lipids were eluted with buffer containing 1% Triton X-100, 50 mM Tris-HCl (pH 7.5) and 300 mM NaCl. Next, the column was washed with buffer A until the disappearance of the optical density of the eluate. IgGs were eluted with 100 mM glycine-HCl buffer (pH 2.6). The obtained fractions of Abs were neutralized with 1 M Tris-HCl buffer (pH 8.8) immediately after leaving the column. After elution of IgGs, the column was washed with 50 mM Tris-HCl (pH 7.5) with 1% Triton X-100 in an amount equal to 10-15 column volumes. Further, the column was washed with 5 mM Tris-HCl (pH 7.5) without detergent in an amount equal to 20-30 column volumes until the protein disappeared in the eluent [42].

#### 2.3.1. Dialysis of IgGs

Dialysis of the isolated IgGs is an additional step of removing low molecular weight substances. Membranes were boiled for 10 minutes in bidistilled water for better opening of the pores immediately before starting dialysis. After that, isolated Abs were dialyzed against 20 mM Na-phosphate buffer (pH 7.0) at +4°C with constant stirring for 17 hours [43, 44].

#### 2.3.2. Determination of Protein Concentration

The protein concentration in Abs was determined using an Epoch spectrophotometer (BioTek, USA). The instrument was calibrated for standard concentration of IgGs. The analysis of the obtained data and determination of the protein concentration were carried out using the software package BioTek Gen5 Software.

#### 2.3.3. Analysis of Electrophoretic Homogeneity of IgGs

SDS-PAGE analysis of IgGs for homogeneity was carried out in 4% to 18% gradient gel. The concentrating gel contained 4% acrylamide, 125 mM Tris-HCl (pH 6.8), and 1% sodium dodecyl sulfate (SDS). The separating gel contained 5-18% acrylamide, 375 mM Tris-HCl (pH 8.8), and 0.4% SDS. IgGs were incubated in a buffer containing 50 mM Tris-HCl (pH 6.8), 2% SDS, 10% glycerin, and 0.025% bromophenol blue at 100°C for 1 min. Thereafter, Abs were loaded into the wells of the gel with 7 *μ*g for each track for Coomassie Blue R-250 staining [45] or 1 *μ*g for each track for silver staining [46]. Electrophoresis was carried out for about an hour at a voltage of 180 V [36]. Gels were washed to a transparent background in the case of Coomassie Blue R-250 staining (2.5 g/l, 10 min, 100°C).

#### 2.3.4. Silver Staining Method

The gel was washed at +22°C for 30 minutes with water to remove SDS after separation of the proteins by electrophoresis. The gel was left overnight in water after incubation for 20-30 minutes in 20% TCA (for fixation) and washing 4 times with water. Then, the gel was kept for 30 minutes in 300 ml of water containing 4-5 mg/ml DTT first and then in 0.1% AgNO_3_ solution in the dark. The gel was rinsed with water and the developer solution of 100 ml twice (6 g of Na_2_CO_3_, 300 ml of water, 140 *μ*l of 40% formalin; formalin was added just before dyeing). Next, the gel was left in 100 ml of developer until the protein bands were clearly visible. After this, the color reaction was stopped with acetic acid (2 ml of glacial acetic acid in 200 ml of water), incubated for 10 minutes, rinsed with water 2-3 times, and left in it for several hours [46, 47].

#### 2.3.5. FPLC Gel Filtration under “Acid Shock” Conditions

100 *μ*l of 1 M glycine-HC1 (pH 2.6) was added to IgGs (0.5 ml, 1.0 mg/ml) of patients with MS and incubated for 30 minutes at room temperature. The gel filtration was performed on a Superdex-200 HR 10/30 column (23 ml) (GE Healthcare Bio-Sciences, Sweden) using ÄKTA pure chromatography system (GE Healthcare Bio-Sciences, Sweden) previously equilibrated with 50 mM glycine-HCl (pH 2.6) containing 0.3 M NaCl. Protein was eluted with the same buffer at a rate of 0.2 ml/min. The release of IgGs from the column was assessed by the changes in optical density of the eluate at 280 nm. The collected fractions were immediately neutralized with 1 M Na-phosphate buffer (pH 8.4) (50 mM Tris-HCl (pH 8.8)). After one week of storage at 4°C for refolding after the “acid shock,” the central part of IgG peaks was used in the SOD activity assays as described below [40].

### 2.4. Determination of Superoxide Dismutase Activity of IgGs

Superoxide dismutase (SOD) activity of IgGs was determined by assessing the degree of inhibition of the reduction of nitroblue tetrazolium (NST) in diformazan superoxide radicals, which are generated during the oxidation of xanthine to uric acid in the presence of xanthine oxidase. The reaction was evaluated on a Lambda 45 spectrophotometer (Perkin Elmer). The reaction was run by adding xanthine oxidase (0.05 U per sample) and 0.1 mM xanthine to the incubation mixture (50 mM sodium carbonate, 0.1 mM EDTA, and 37.5 *μ*M nitroblue tetrazolium (NBT) in 50 mM phosphate buffer (pH 10.2)).

The initial rate of NBT recovery without SOD sample activity per minute was determined by the change in optical density of the solution (*E*_1_), which was recorded by a kinetic curve for 5–7 min at a wavelength of 560 nm. A sample containing purified IgGs was added to the same cuvette, and change in the optical density of the solution per minute (*E*_2_) was recorded for 5 minutes in the presence of IgGs with SOD activity. A sample of IgGs was added to the cuvette in an amount that resulted in a change in optical density of 0.017–0.020 U per minute under conditions of an excess of substrate for proportional change in the rate of the recorded reaction.

SOD activity of IgGs was calculated by the formula (*Е*_1_ − *Е*_2_) × (2150 + *V*)/3 × 10^−2^ × *V* × *C* = *U* (*μ*M diformazan/min/mg of protein), where *U* is the unit of enzyme activity, 2150 *μ*l is the volume of the incubation medium, 3 × 10^−2^ is the molar extinction coefficient of diformazan, *V* is the sample volume, *С* is the concentration of IgGs (mg/ml), *E*_1_ is the change in the optical density of the solution per minute that reduces NBT to diformazan in the absence of SOD activity, and *E*_2_ is the change in the optical density of the solution per minute that reduces NBT to diformazan by superoxide in the presence of IgGs with SOD activity.

The unit of SOD activity (*U*) was taken as the difference in the amount of reduced diformazan without the participation of SOD, and the amount of diformazan reduced when this reaction was inhibited by SOD per minute in 1 ml of solution per 1 mg of protein in the sample (*μ*M diformazan/min/mg of protein) [48].

### 2.5. Estimation of the Kinetic Parameters of SOD Activity of IgGs

NBT concentrations of 2 *μ*M, 5 *μ*M, 10 *μ*M, 20 *μ*M, 35 *μ*M, and 50 *μ*M were used to determine the kinetic parameters of the *K*_m_, *V*_max_, and *K*_cat_ SOD reaction of IgGs. The values of apparent *K*_m_ and *V*_max_ (*k*_cat_) were calculated based on the rate of superoxide dismutation and NBT concentration. The *K*_m_ and *V*_max_ values were estimated using the nonlinear regression method in the Origin Pro v.8.6 program and were presented as linear transformations using a Lineweaver-Burk plot. The value of *K*_cat_ was determined by the formula *K*_cat_ = *V*_max_/[AB], where [AB] is the total concentration of IgGs in the reaction mixture [40, 42, 49].

### 2.6. Quantitative Inhibition of SOD Reaction of IgGs

The use of inhibitory analysis is of great interest for elucidating the mechanism of catalysis. For this purpose, substances blocking certain groups of the active catalytic center were used. Evaluation of the degree of inhibition of the catalytic activity of the SOD reaction of IgGs in patients with MS was performed using both specific and nonspecific inhibitors.

#### 2.6.1. Inhibitory Analysis Using Triethylenetetramine

A specific SOD enzyme inhibitor triethylenetetramine (TETA) was used as a SOD inhibitor of the IgG activity at concentrations of 10 mM, 15 mM, 20 mM, and 50 mM. The SOD activity of IgGs without inhibitor was determined and in the presence of the listed inhibitor concentrations in the reaction mixture. The results were presented as percentages. SOD activity of IgGs without inhibitor was taken as 100% [50, 51].

#### 2.6.2. Inhibitory Analysis Using EDTA

The mechanism of action of EDTA (ethylenediaminetetraacetate), which is a nonspecific inhibitor of enzymatic activity, is based on the binding of divalent metal ions. EDTA inhibitor was used at a concentration of 50 mM [52]. Superoxide dismutase activity of IgGs was determined without an inhibitor and in the presence of EDTA in the reaction mixture. The results were presented as percentages. SOD activity of IgGs without inhibitor was taken as 100%.

### 2.7. Determination of Catalase Activity of IgGs

The catalase activity of IgGs was determined out according to Aebi [53]. The reaction was run by adding 100 *μ*l IgGs (concentration 0.5 mg/ml) to the incubation mixture (50 mM potassium phosphate (pH 7.0), 30 mM H_2_O_2_). The catalase activity of IgGs was determined spectrophotometrically from a decrease of absorbance at 240 nm for 5-8 min caused by the splitting of hydrogen peroxide using Lambda 45 spectrophotometer (Perkin Elmer). All measurements were taken within the linear regions of the time courses and linear part of relative activity dependence upon IgG concentration. For the calculation of the activity, the molar extinction coefficient of hydrogen peroxide (*ε* = 0.081 mM^−1^/cm^−1^) was used [53]. The measured relative activities for IgGs were normalized to standard conditions (mM Н_2_О_2_/min/mg IgGs).

### 2.8. Estimation of the Kinetic Parameters of Catalase Activity of IgGs

H_2_O_2_ concentrations of 15 mM, 20 mM, 25 mM, 30 mM, 35 mM, 40 mM, and 45 mM were used to determine the kinetic parameters of the *K*_m_, *V*_max_, and *K*_cat_ catalase reaction of IgGs. The values of apparent *K*_m_ and *V*_max_ (*k*_cat_) were calculated based on the rate reduction of the concentration of H_2_O_2_ depending on the concentration. The *K*_m_ and *V*_max_ values were estimated using the nonlinear regression method in the Origin Pro v.8.6 program and were presented as linear transformations using a Lineweaver-Burk plot [54]. The value of *K*_cat_ was determined by the formula *K*_cat_ = *V*_max_/[AB], where [AB] is the total concentration of IgGs in the reaction mixture [40, 42, 49]. Errors in the values were within 7-15%. The results are reported as median of at least three independent experiments for each sample of IgGs.

### 2.9. Statistical Analysis

Statistical data analysis was executed using the Statistica version 10.0 (StatSoft, Tulsa, OK, USA). The hypothesis of normal distribution was tested using Shapiro-Wilk criterion for all available data samples. Nonnormally distributed quantitative data were presented as medians and quartiles (Q1-25 and Q-75%). The Kruskal-Wallis one-way analysis of variance (ANOVA) and median test were used for data comparison between three groups. Mann–Whitney *U* test was used to assess for significant differences between two groups. A *P* value  <  0.05 was deemed to be statistically significant.

## 3. Results and Discussion

### 3.1. Purification and Characteristics of IgGs and Application of Strict Criteria

According to modern concepts, the assignment of the studied catalytic activity directly to IgGs requires the verification of several stringent criteria [55] such as the use of specific affinity sorbents for the isolation of Abs, the homogeneity of the obtained Abs in electrophoretic analysis with a gradient polyacrylamide gel, and maintenance of catalytic activity after high-performance gel-filtration chromatography under pH shock conditions.

**Figure 1:**
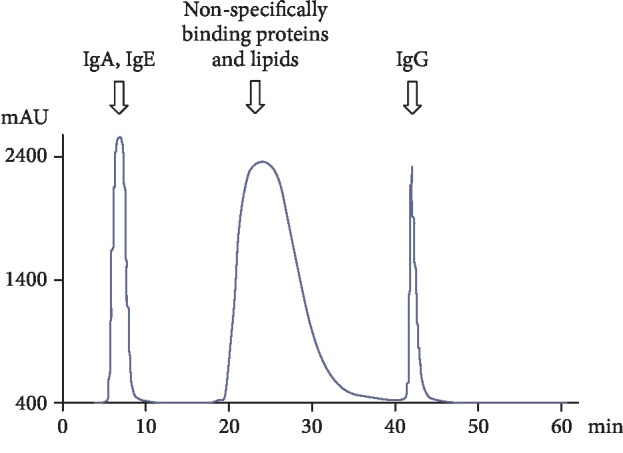
Profile affinity chromatography (ÄKTA pure, GE) of IgGs isolated from the serum of patients with MS on a column with immobilized protein G.

The fulfillment of the first one of these criteria of the attribution of catalytic activity to Abs is the release of IgGs using specific affinity sorbents. It is presented in the form of a profile of affinity chromatography (ÄKTA pure, GE) IgGs from the serum of patients with MS on a column with immobilized proteins G (Figure 1).

The affinity chromatography on carriers with immobilized G proteins of Staphylococcus aureus was used to isolate IgGs. Protein G is able to specifically bind Fc fragments of all IgG subclasses, thereby providing a highly selective isolation of the corresponding class of immunoglobulins. We used HiTrap Protein G HP columns with Protein G Sepharose. The sorbents were washed with buffer containing 1% Triton X-100 and 0.3 M NaCl after adsorption of the antibodies to destroy possible noncovalent complexes of Abs with cellular proteins and to remove the lipid fraction. Elution of specifically adsorbed fractions of Abs was performed with 0.1 M glycine-HCl buffer (pH 2.6). Thus, the possibility of isolating additional protein impurities was excluded.

**Figure 2:**
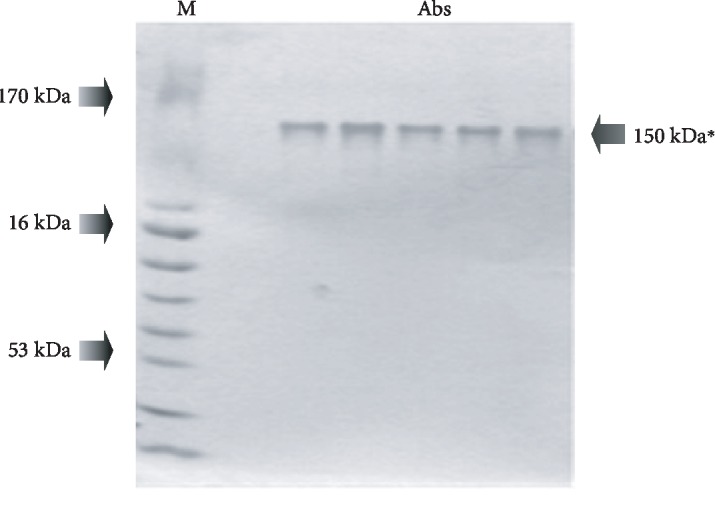
Electrophoretic analysis of the homogeneity of Abs after AgNO_3_ staining. M: protein molecular weight markers, Abs: studied IgGs. Note: ^∗^150 kDa band corresponds to IgGs on electrophoregram.

The second criterion for attributing SOD activity directly to Abs is the electrophoretic homogeneity of IgGs with characteristic molecular weight. This was confirmed by gradient gel electrophoresis in a polyacrylamide gel (4–18%) followed by silver staining. The presence of a single band was shown for the lane of molecular weight 150 kDa (Figure 2).

Another important criterion for attributing the observed catalytic activity to Abs is the gel filtration of IgGs carried out under acidic conditions. Gel filtration at acidic pH values provides dissociation and subsequent separation of all components of immunocomplexes consisting of immunoglobulins of different classes, which can bind with different antigens. At this stage, classical enzymes are subjected to acid shock, leading to complete and irreversible loss of their activity in many cases. Since the molecular weights of enzymes differ from the molecular weight of IgGs, it is possible to establish which of the proteins the catalytic activity belongs to. The stability of any abzymes to these conditions is relatively low, so their activity is significantly reduced immediately after gel filtration. It has been previously shown that activity of IgGs was restored after their neutral solutions were kept at 40°C for 1-2 weeks [40, 42].

**Figure 3:**
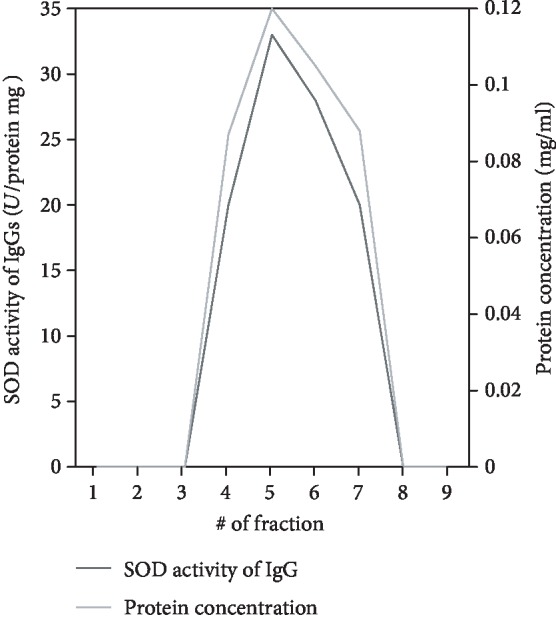
Protein elution profile of FPLC gel filtration of IgGs in pH shock conditions (red line) and SOD activity profile of IgGs in the obtained fractions (blue line). Only part of fractions corresponding to specific elution of IgGs is shown in patients with MS. *U*: *μ*M of diformazan/min.

Analysis of SOD activity of IgGs from blood serum in the control group and in groups of patients with MS showed that the activity remained after gel filtration under the conditions of acid shock only in fractions corresponding to the optical density profile (A280) of Abs. Activity was not detected in the remaining fractions. Protein profiles of FPLC gel filtration of IgGs after incubation under pH shock conditions (1 M glycine-HCl (pH 2.6)) and elution with 50 mM glycine-HCl (pH 2.6) and 0.3 M NaCl repeated SOD activity profiles of IgGs in the obtained fractions in all groups. A graphical comparison of the protein elution profile in gel filtration and the SOD profile of IgG activities in the obtained fractions is shown in Figure 3. Thus, proof of the SOD activity belonging to the Abs is the overlay of the SOD activity maximum and the top of the chromatographic peak corresponding to the IgGs.

Hence, we showed that, based on generally accepted stringent criteria for attributing observed catalytic activity to Abs, the studied SOD activity is a property of the studied IgGs.

### 3.2. Superoxide Dismutase Activity of IgGs in Patients with Different Types of Multiple Sclerosis

**Figure 4:**
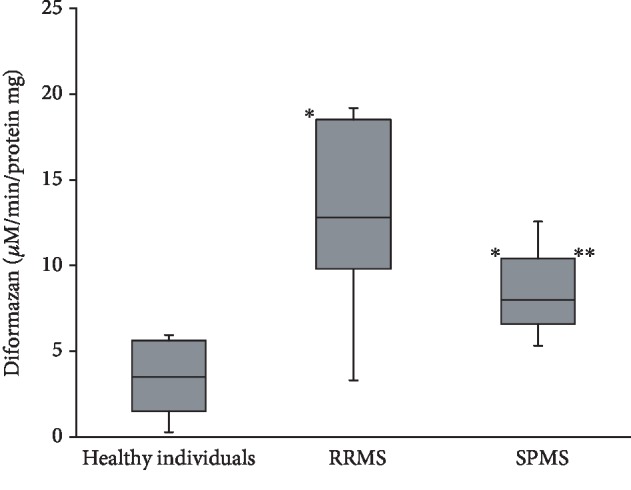
SOD activity of IgGs in patients with RRMS and SPMS and healthy individuals. Note: ^∗^*P* < 0.05 compared with healthy donors. ^∗∗^*P* < 0.05 compared with patients with RRMS (Me [*Q*_25_; *Q*_75_]).

In this work, we first evaluated the SOD activity of IgGs in healthy individuals and patients with different types of MS. The SOD activity of IgGs was investigated using a spectrophotometric method to determine the degree of inhibition of the reduction of nitroblue tetrazolium to diformazan by superoxide radicals generated by the system of enzymatic oxidation of xanthine to uric acid in the presence of xanthine oxidase. SOD activity levels of IgGs in the studied groups are presented in Figure 4. The results of the study showed that SOD activity of IgGs in patients with MS was almost 2-4 times higher as compared to healthy individuals (*P* < 0.05). The highest SOD activity levels of IgGs (12.8 [9.8–18.5] *μ*M diformazan/min/mg) in patients with RRMS was 4 times higher than those in healthy individuals (3.5 [1, 5-5.6] *μ*M of diformazan/min/mg of protein) (*P* = 0.002) and 2 times higher than those in patients with SPMS (8.0 [6.6-10.4] *μ*M mol of diformazan/min/mg of protein) (*P* = 0.018). The SOD activity of IgGs in patients with SPMS was twice higher than that in healthy individuals (*P* = 0.011).

The canonical enzyme SOD belongs to the class of oxidoreductases. Its only function is catalysis of the dismutation of superoxide radicals; therefore, it prevents the transformation of the superoxide anion radical into a more toxic hydroxyl radical in the cell. Thus, SOD markedly inhibits lipid and protein peroxidation. It is mainly an intracellular enzyme, and only a small part of SOD activity was detected by Marklund et al. in mammalian extracellular fluids in the form of glycosylated tetramer copper, zinc-SOD [56].

The results of numerous studies convincingly prove that large amounts of ROS are continuously formed in patients with MS [11, 12]. In addition, according to our previous studies, SOD activity in the erythrocytes of patients with MS is 2-3 times lower in comparison to healthy subjects [13]. Thus, it is possible that Abs with high levels of SOD activity in various forms of MS significantly exceeding the activity levels in healthy individuals compensate for the low activity of the SOD in this disease. It is assumed that these abzymes are able to protect neurons and their axons from free-radical damage reducing the excess amount of superoxide, i.e., catalytic Abs are an additional factor that strengthens the AODS. The increased SOD activity levels of IgGs in MS can be considered as a protective anti-inflammatory mechanism in the extracellular environment.

It is important to note that catalytic SOD activity level of IgGs decreases twice with the increase in the severity of the pathological process (in patients with SPMS in comparison with patients with RRMS) but it is two times higher than the physiological norm.

The data obtained are possibly related to the biphasic development of MS. The highest SOD activity level of IgGs in patients with RRMS may be associated with the initial phase of the disease characterized by predominance of immune inflammation processes and high activity of the humoral immune response and, as a result, increased production of Abs. It can be assumed that the level of catalytic SOD activity of IgGs decreases with the progression of the disease and its transition to the second neurodegenerative phase characterized by inhibition of B cell immunity. It is possible that an increase in the levels of uncompensated OS in patients with SPMS is related to the occurrence of the most pronounced neurological abnormalities and, accordingly, an increase in the degree of disability.

### 3.3. Determination of the Kinetic Parameters of SOD Reaction of IgGs

**Figure 5:**
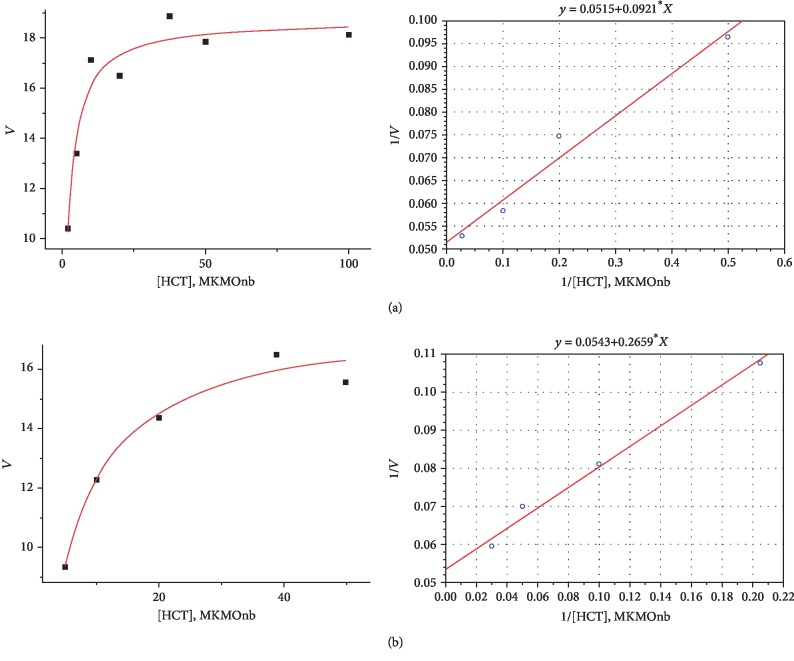
Dependencies of relative rates of SOD activity of IgGs on the concentration of NBT and determination of the *K*_m_ and *V*_max_ (*k*_cat_) values. 1: hyperbolic curve in *V* coordinates from *S*; 2: graph plotted in inverse coordinates 1/*V* from 1/*S* using the Lineweaver-Burk plot. (a) IgG #1 from a healthy donor; (b) IgG #2 from a patient with SPMS.

We have estimated the *K*_m_ for NBT and *V*_max_ (*k*_cat_) values using the following two samples of IgGs: IgG #1 from a healthy individual (Figure 5(a)) and IgG #2 from a patient with SPMS (Figure 5(b)). The values of *K*_m_ and *V*_max_ (*k*_cat_) were calculated from the dependencies of dismutation rate of the superoxide radical on the concentration of NBT. The dependence of the superoxide dismutation rate on the NBT concentration is presented in the figures in two coordinate systems of the direct (1) and inverse (2) coordinates. The error of the initial rate determination from three experiments in every case did not exceed 7 ± 10%.

In our study, it was found that there are no significant differences for such kinetic parameters as the initial (instantaneous) speed (*V*) and maximum speed (*V*_max_) of the SOD reaction of IgGs, unlike a large difference in the specific SOD activity of IgGs between patients and healthy individuals. As can be seen in Table 1, both samples of IgGs are characterized by similar kinetic reaction parameters. It is also necessary to note that the IgG #2 from the patient with SPMS is characterized by a lower *K*_m_ value (numbers) and, accordingly, has a greater affinity for the substrate when compared with that of a healthy individual.

When analyzing the obtained results (Table 1), we see that the *K*_m_ value of SOD activity of IgGs is significantly lower (70 times) than *K*_m_ in erythrocyte SOD [57]. This indicates a greater affinity for the abzyme to the substrate. As a result, a sufficiently strong AB-substrate complex is formed with a dissociation that takes a lot of energy and time. Therefore, IgG with SOD activity has a lower rate of catalysis compared with the true enzyme. This is also evidenced by the value of the catalytic constant (*K*_cat_) which has similar values in samples IgG #1 and IgG #2. *K*_cat_ in erythrocyte SOD is 5-6 orders of magnitude higher [57].

### 3.4. Inhibitory Analysis of Superoxide Dismutase Activity of IgGs

**Figure 6:**
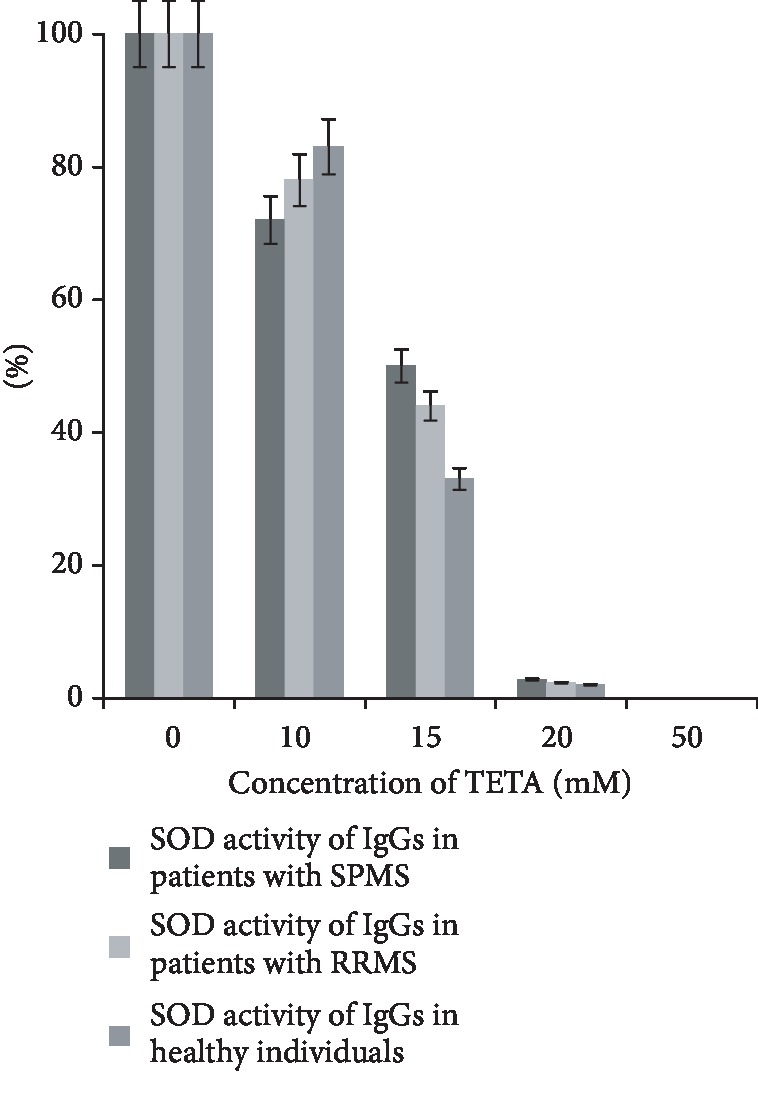
The dependence of SOD activity of IgGs on the concentration of TETA. The error bars represent the standard deviation of measurements for SOD activity in five separate sample runs (*n* = 65).

Triethylenetetramine (TETA), a specific superoxide dismutase enzyme inhibitor, was used for inhibitory analysis. Inhibitor concentrations of 10 mM, 15 mM, 20 mM, and 50 mM were used. Results were presented as percentages. SOD activity without inhibitor was taken as 100% (Figure 6).

TETA is a compound capable of forming a chelate complex with copper [58]. Inhibitory analysis showed that TETA inhibits SOD activity of IgGs starting with 10 mM concentration. It should be noted that the inhibitory effect of a specific inhibitor TETA on IgGs was dependent on the type of MS. For example, the maximum inhibition of SOD activity of IgGs was observed in the group of healthy individuals, while the minimum was observed in the group of patients with SPMS at a TETA concentration of 15 mM. The difference between these two groups was statistically significant. This result is probably indicative of the conformational differences of IgGs in different groups. A 50 mM TETA solution completely inhibits SOD activity in all three groups. Consequently, there is an assumption regarding the participation of copper ions in the catalysis of this reaction by IgGs.

**Figure 7:**
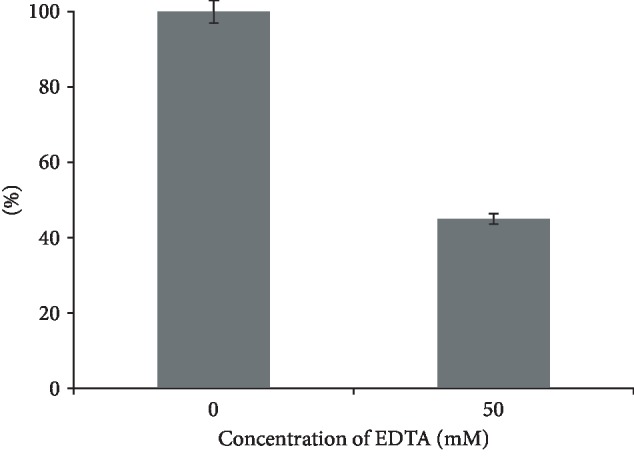
Dependence of SOD activity IgGs in patients with SPMS on the EDTA concentration. The error bars represent the standard deviation of measurements for SOD activity in five separate sample runs (*n* = 20).

We inhibited SOD activity by a nonspecific EDTA inhibitor with a concentration of 50 mM to test the assumption regarding the participation of metal ions in the mechanism of reactions catalyzed by IgGs. The inhibitor analysis was performed in Abs of patients with SPMS. Results were presented as percentages; activity without inhibitor was considered 100% (Figure 7).

The mechanism of the inhibitory action of EDTA is due to the binding of divalent metal ions. Figure 7 shows that EDTA at a concentration of 50 mM inhibits the SOD activity of IgGs by 45%. Inhibition of the studied catalytic activity of IgGs by both specific and nonspecific inhibitors confirms the hypothesis that Abs are associated with divalent metal ions. This is also consistent with the already known data on the study of the oxidoreductase activity of IgGs [30, 36]. In this regard, more detailed studies are needed to confirm and provide further details of this phenomenon.

### 3.5. Catalase Activity and Kinetic Parameters of IgGs in Patients with Different Types of Multiple Sclerosis

**Figure 8:**
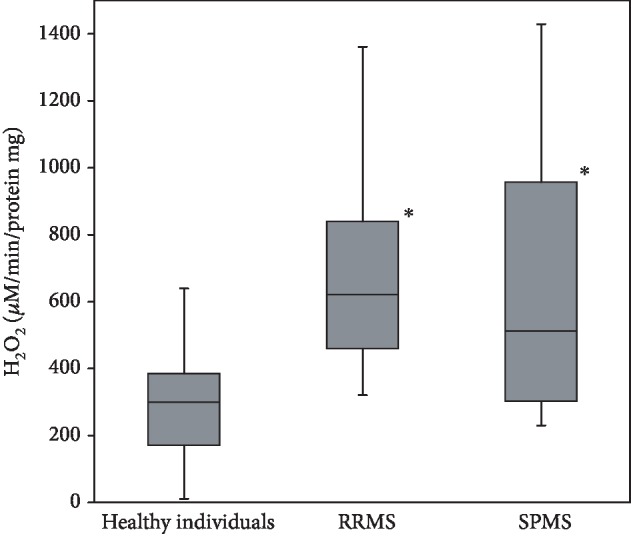
Catalase activity of IgGs in patients with RRMS and SPMS and healthy individuals. Note: ^∗^*P* < 0.05 compared with healthy donors (Me [*Q*_25_; *Q*_75_]).

Due to the fact that catalase follows after SOD pathway and it completes this process, we first evaluated the catalase activity of IgGs in healthy individuals and patients with different types of MS. Catalase activity levels of IgGs in the studied groups are presented in Figure 8. The results of the study showed that catalase activity of IgGs in healthy individuals (300.3 [171.1–384.8] *μ*M of H_2_O_2_/min/mg of protein) was almost 2 times lower as compared to that of patients with RRMS (621.5 [460.0–840.0] *μ*M of H_2_O_2_/min/mg of protein) (*P* = 0.001) and that of patients with SPMS (513.0 [303.2–957.0] *μ*M of H_2_O_2_/min/mg of protein) (*P* = 0.009). No differences were found between patients with different types of MS.

When analyzing the kinetic parameters of IgG from the patient with SPMS, we see that the *K*_m_ value of catalase activity of IgGs is significantly lower (10^4^ times) than *K*_m_ in erythrocyte catalase (36.6 [4, 8–45] *μ*M and 13.9∗10^4^ *μ*M, respectively) [59]. The *K*_cat_ value of catalase activity of IgGs is 10^8^ times lower than *K*_cat_ in erythrocyte catalase (0.205 [0.178-0,232] min^−1^ and 0.222∗10^8^ min^−1^, respectively) [59]. Thus, when analyzing the kinetic parameters of the catalase reaction of IgGs in patients with multiple sclerosis, the *K*_m_ values obtained were significantly lower than those of classical enzymes. This indicates a greater affinity of abzymes for the substrate, which is quite natural for Abs. As a result of this, a stronger enzyme-substrate complex is formed, the dissociation of which takes more time; therefore, IgGs have a lower catalysis rate compared to enzymes.

## 4. Conclusions

Overall, our work shows for the first time that IgGs isolated from the blood serum of patients with MS and healthy individuals have the ability to catalyze the dismutation reaction of the superoxide anion radical. We also showed for the first time that IgGs of patients with MS are capable of degrading hydrogen hydroperoxide. SOD and catalase activity of IgGs of patients with MS is significantly increased in comparison to that of healthy donors. This activity is an intrinsic property of Abs, which is confirmed by a number of stringent criteria such as the isolation of immunologically and electrophoretically homogeneous polyclonal IgGs on Protein G Sepharose, followed by gel filtration in acidic conditions destroying immune complexes.

The pathogenesis of MS is still not fully understood, and there are open questions about the factors that determine the type of disease, symptoms, rate of disease progression, and effectiveness of therapy. The two most common types of MS, RRMS and SPMS, which have been included in this study, often lead to disability and a significantly reduced quality of life for the patient. The SOD activity of IgGs in patients with RRMS is 4 times higher than that in healthy individuals, while in patients with SPMS it is two times higher than in healthy subjects. The kinetic characteristics of the SOD reaction of IgGs are several orders of magnitude lower than those of the SOD enzyme itself. Consequently, abzymes with SOD activity have a lower catalysis rate compared with that of the enzyme. The studied activity is inhibited by classical metal-dependent inhibitors EDTA and TETA. Based on the results of this study, it can be assumed that abzymes with SOD and catalase activity are involved in binding and removing ROS from the bloodstream, thereby protecting the body from oxidative stress. However, further studies will be needed to determine the contribution of Abs with oxidoreductase activity to the pathogenesis of MS.

## Figures and Tables

**Table 1:** Comparative characteristics of the kinetic parameters of human SOD activity of IgGs and the kinetic parameters of SOD activity in human erythrocytes.

Parameters	IgG #1 (from healthy individual) Me [*Q*_25_; *Q*_75_]	IgG #2 (SPMS) Me [*Q*_25_; *Q*_75_]	SOD^∗^ in erythrocytes
*K*_m_ (*μ*M)	4.69 [4.49; 4.89]	1.2 [1.16; 1.78]	360
*V*_max_ (*μ*mol of diformazan/min)	18.1 [17.8; 18.4]	18.95 [18.8; 19.1]	
*K*_cat_ (min^-l^)	6. 82 [6.74; 6.96]	5.73 [5.69; 5.78]	1 × 10^6^

^∗^Data are taken from the literature [55].

## Data Availability

Data used to support the findings of this study are available from the corresponding author upon request.
